# T151. APATHY AND DIMINISHED EXPRESSION ARE NOT ASSOCIATED WITH VENTRAL OR DORSAL STRIATUM VOLUME IN SCHIZOPHRENIA

**DOI:** 10.1093/schbul/sby016.427

**Published:** 2018-04-01

**Authors:** Achim Burrer, Matthias Kirschner, Erich Seifritz, Kaiser Stefan

**Affiliations:** 1 University Hospital of Psychiatry, University of Zurich; 2 Geneva University Hospitals

## Abstract

**Background:**

Negative symptoms are core features of schizophrenia and can be grouped into two domains. These are apathy including anhedonia, avolition and asocialty as well as diminished expression including blunted affect and alogia. A large body of research found that ventral striatal hypoactivation is linked to negative symptoms. In particular, it has been shown that this neural correlate is specific for apathy but not diminished expression. Here, we investigated whether this dissociation can also be found in ventral striatum volume.

**Methods:**

We included brain structural T1 MRI data of 60 patients diagnosed with schizophrenia (SZ) and 58 healthy controls (HC). Negative symptoms in these groups have been assessed using the Brief Negative Symptom Scale (BNSS). We performed voxel-based morphometry (VBM) using the statistical parametric mapping package (SPM 12; Wellcome Trust Centre for Neuroimaging, London). We performed a region of interest (ROI) analysis of ventral and dorsal striatal volume between patients with schizophrenia and healthy controls. Furthermore, we analyzed the correlation of right and left ventral striatal volume with apathy and diminished expression in patients with schizophrenia. Moreover, we analyzed potential group differences in gray matter volume in an exploratory whole-brain analysis. Finally, we performed an exploratory whole-brain linear regression to identify potential correlations between the two negative symptom dimensions and gray matter volume. (cluster-defining threshold of p < 0.001, cluster-level pFWE < 0.05)

**Results:**

Patients with schizophrenia showed no differences in ventral striatal volume compared to healthy controls. Apathy or diminished expression did not correlate with ventral or dorsal striatal gray matter volume in patients with schizophrenia. In the exploratory whole-brain analysis we found significant less gray matter volume in the right insula of schizophrenia patients compared to healthy controls (cluster-level pFWE = 0.03, peak (x,y,z = 46,-15,20). Our exploratory whole-brain linear regression revealed no significant correlation between apathy or diminished expression and gray matter volume changes in patients with schizophrenia.

**Discussion:**

Although a correlation of apathy and ventral striatal volume has been shown in a previous study with fewer subjects, we could not reproduce this finding in a larger group of 60 patients with schizophrenia (Roth et al. 2016). However, while these negative findings do not support the association between apathy and ventral striatal volume, there may be more subtle brain structural changes linked to the pathophysiology of apathy, which cannot be detected by voxel based morphometry. The gray matter reduction in the right insula in subjects with schizophrenia replicated findings from previous studies in schizophrenia (Fornito et al. 2009).

